# Challenges in Emerging and Reemerging Arboviral Diseases: The Examples of Oropouche and Yellow Fever

**DOI:** 10.3390/pathogens14070621

**Published:** 2025-06-23

**Authors:** Alfonso J. Rodriguez-Morales, Ranjit Sah, Carlos Ramiro Silva-Ramos, Diana Marcela Pava-Garzón

**Affiliations:** 1 Faculty of Health Sciences, Universidad Científica del Sur, Lima 15067, Peru; 2 Grupo de Investigación Biomedicina, Facultad de Medicina, Fundación Universitaria Autónoma de las Américas-Institución Universitaria Visión de las Américas, Pereira 660003, Risaralda, Colombia; 3 Department of Medicine, Dr. D. Y. Patil Medical College, Hospital and Research Centre, Dr. D. Y. Patil Vidyapeeth (Deemed-to-be-University), Pimpri, Pune 411018, Maharashtra, India; ranjitsah57@gmail.com; 4 SR Sanjeevani Hospital, Kalyanpur-10, Siraha 121102, Nepal; 5 Grupo de Enfermedades Infecciosas, Departamento de Microbiología, Facultad de Ciencias, Pontificia Universidad Javeriana, Bogotá D.C. 110311, Colombia; cramiro-silva@javeriana.edu.co; 6 Instituto Nacional de Salud (INS), Bogotá D.C. 111321, Colombia; dpava@ins.gov.co

The global burden of arboviral diseases continues to rise with alarming frequency and impact [1]. Traditionally associated with tropical and subtropical regions, arboviruses are increasingly becoming a public health concern in broader geographical areas due to urbanization, climate change, deforestation, and increased human mobility [2]. Among these, Oropouche virus (OROV) and yellow fever virus (YFV), recently renamed as *Orthobunyavirus oropoucheense* and *Orthoflavivirus flavi*, respectively [3,4], represent two distinct yet illustrative examples of the complex and evolving challenges posed by emerging and reemerging arboviruses [5,6,7]. Recent and ongoing outbreaks of both viruses highlight significant gaps in surveillance, diagnostics, clinical management, and prevention—gaps that must be urgently addressed to strengthen global preparedness and responses [8,9,10]. In 2024 and 2025, OROV gained attention due to a marked increase in reported cases [11,12]. YFV remains a significant concern due to its high case fatality rate (Table 1).

Oropouche virus, an orthobunyavirus transmitted by *Culicoides paraensis* midges, remains a neglected tropical arbovirus despite its frequent outbreaks in South America [13,14,15]. Since its first identification in Trinidad and Tobago in 1955, OROV has caused numerous epidemics, particularly in the Amazonian regions of Brazil, Peru, and, more recently, Ecuador and Colombia [16]. As part of the broad differential diagnosis of acute undifferentiated febrile illness, OROV is clinically indistinguishable from other etiologies, often leading to misdiagnosis and underreporting, which complicates its recognition and documentation [12]. Although generally self-limiting, OROV infection can be debilitating and, in some cases, associated with neurologic complications such as meningitis and encephalitis [17,18].

The lack of awareness, coupled with minimal surveillance infrastructure and the absence of point-of-care diagnostics, means that the actual burden of Oropouche virus (OROV) remains vastly underestimated. In the absence of a vaccine, clinical suspicion plays a crucial role; however, it is currently insufficient due to limited understanding of the disease and its inconsistent inclusion in differential diagnoses by physicians. Most cases remain undetected or misclassified, as there are no validated rapid diagnostic tests or field-deployable serological assays. Confirmatory testing depends on RT-PCR, which is primarily performed in centralized laboratories using standardized protocols across several countries. While this facilitates regional monitoring, it often leads to significant delays in outbreak detection and response. Expanding RT-PCR capabilities to subnational laboratories remains a critical yet challenging goal that could strengthen early detection. This persistent diagnostic gap, combined with OROV’s potential for urban outbreaks due to its vector ecology, constitutes a latent but serious public health threat [19,20].

In contrast, yellow fever is a well-known, historically devastating flavivirus that continues to pose a significant challenge to public health systems in endemic regions [21]. While a safe and effective vaccine exists and is part of routine immunization programs in many countries [8], the past decade has witnessed a resurgence of yellow fever, with significant outbreaks occurring in Angola, Brazil, Nigeria, and, more recently, Colombia [22]. These events highlight critical vulnerabilities in vaccination coverage for areas with difficult geographical access and outbreaks [11,23,24].

A particularly concerning aspect of the resurgence of yellow fever in the Americas has been its encroachment into previously unaffected areas, including urban and peri-urban zones, raising fears of large-scale outbreaks [25,26]. In Colombia, severe yellow fever cases in humans [27], as well as confirmed infections in non-human primates (NHPs) [28], have underscored the importance of sylvatic surveillance and the need for rapid, evidence-based clinical management protocols [29]. Encouragingly, recent developments such as the proposed clinical algorithm for managing severe yellow fever in Colombia provide a valuable step forward, aligning clinical decision-making with the available evidence [29]. However, additional tools are needed, which must be updated continuously as new evidence emerges.

Despite the availability of the yellow fever vaccine (Table 1), global production remains insufficient, particularly in response to unexpected outbreak demands [30,31]. The World Health Organization (WHO)’s adoption of fractional dosing as an emergency strategy—using one-fifth of the standard dose—has stirred both praise for its practicality and concern regarding the duration of immunity conferred, particularly in children [30,31,32,33,34]. This situation highlights the broader issue of vaccine supply chain fragility, even for long-standing diseases with established preventive measures in place.

Both OROV and YFV illustrate fundamental shortcomings in arboviral disease surveillance. For OROV, weak or absent surveillance networks in some countries, particularly outside South America [35], lead to underreporting, which hinders the development of effective public health interventions [12]. For YFV, surveillance often hinges on the detection of sentinel events such as NHP die-offs, which, while valuable, are reactive rather than proactive [28].

Another major limitation is the scarcity of robust, field-deployable diagnostic tools. For OROV, the lack of serological assays remains a critical barrier to case confirmation, particularly in remote or resource-limited settings (Table 1). YFV faces a different but equally challenging situation: while serological tests exist, they often cross-react with other flaviviruses, particularly in dengue- and Zika-endemic regions, which complicates their interpretation (Table 1).

Here, genomic epidemiology emerges as a powerful tool with transformative potential [36,37,38]. By enabling the high-resolution tracking of viral evolution, lineage displacement, and transmission dynamics, genomic surveillance can guide targeted interventions and monitor vaccine efficacy (Table 1) [27]. The integration of sequencing data with traditional epidemiological methods should become standard practice, especially as costs decrease and portable sequencing platforms become more accessible. However, implementation of these tools in real time and hypotheses formulation remains a challenge, as the measurement of these genetic indicators will always be limited by the amount of viral population analyzed, the occurrence of cases in each region, as well as the biological and genetic characteristics of each virus.

The development of evidence-based clinical guidelines for severe arboviral diseases remains uneven. While the recent proposal for yellow fever management in Colombia marks essential progress [29], there is a lack of guidelines for OROV. In 2024, the Committee on Tropical Medicine of the Pan American Association of Infectious Diseases developed recommendations for diagnostic approaches toward and the clinical and epidemiological management of patients with suspected Mayaro and Oropouche virus infections. Unfortunately, these recommendations are only available in Spanish and can only be accessed on their website (https://apiinfectologia.org/wp-content/uploads/2024/04/Mayaro_Oropouche-V2.pdf) (accessed on 1 April 2025).

Given the potential for severe neurological complications [39,40,41] and the burden of misdiagnosed febrile illness, developing clinical algorithms for OROV management, even if initially empirical, should be prioritized. Prospective clinical studies, supported by improved diagnostics, will be key to this endeavor.

Prevention remains the cornerstone of arboviral disease control. For YFV, while the vaccine is effective, some challenges persist, including coverage gaps, logistical hurdles, and unresolved concerns about vaccine safety and fractional dosing. Strategic investment in vaccine production capacity, stockpile management, and targeted immunization campaigns is crucial for ensuring preparedness and a rapid response. In contrast, the OROV situation is far more concerning. There are currently no licensed vaccines, and candidate vaccines are far from implementation, still in the preclinical stages, without ongoing human trials [42,43]. The lack of commercial interest, despite recurring outbreaks and an expanding geographic range, reflects the broader neglect of “orphan” arboviruses. Public–private partnerships, facilitated by international health organizations, will be critical in advancing vaccine development for OROV and similar emerging threats. Accelerating vaccine development for OROV and other emerging threats is not only a scientific necessity, but also a global public health imperative that demands sustained political will, strategic investment, and coordinated public–private partnerships, actively led by international health organizations, to avoid repeating cycles of neglect and unpreparedness in the face of the next epidemic.

The concurrent emergence and reemergence of OROV and YFV illustrate the multifaceted challenges that come with arboviral diseases in the 21st century. While yellow fever serves as a cautionary tale of resurgence despite existing preventive tools, OROV reminds us of the threats posed by under-recognized and under-researched viruses. Both demand urgent attention to strengthen surveillance, improve diagnostics, promote genomic epidemiology, and develop evidence-based clinical management strategies. Ultimately, sustained investment, international collaboration, and political commitment will be necessary to close these critical gaps and enhance global health security.

## Figures and Tables

**Table 1:** Main epidemiological parameters for comparing Oropouche and Yellow Fever in the Americas during the 2024/2025 epidemics.

Variable	Oropouche	Yellow Fever
Cases in 2024/2025	28,320	295
Deaths	9	125
Case Fatality Rates (%)	0.03	42.37
Affected Countries in the Region	14	6
Availability of Vaccine	No	Yes (17D vaccine)
Availability of Field Serological Test	None available	Limited (cross-reactivity with flaviviruses)
Evidence-Based Clinical Guidelines Published in Peer-Reviewed Journals	Not available	Available (e.g., Colombia, 2025)
Main Vectors	*Culicoides paraensis*	Sylvatic cycle: *Haemagogus*, *Sabethes* spp.
Genomic Surveillance Implementation	Limited	Increasingly applied
Main Reservoir Host	Unknown (possibly sloths, primates)	Non-human primates
